# Ectopic expression of MELK in oral squamous cell carcinoma and its correlation with epithelial mesenchymal transition

**DOI:** 10.18632/aging.202986

**Published:** 2021-05-07

**Authors:** Bo Li, Xinghuanyu Xu, Xin Bin, Jiang Zhou, Zhangui Tang

**Affiliations:** 1 Xiangya School of Stomatology, Central South University, Changsha, Hunan 410008, PR China

**Keywords:** MELK, EMT, OSCC

## Abstract

Epithelial–mesenchymal transition (EMT) is closely correlated to metastasis formation generation and maintenance of cancer stem cells, nevertheless, the underlying mechanisms are unclear. The aim of this study is to investigate the role of maternal embryonic leucine-zipper kinase (MELK) in EMT regulation in oral squamous cell carcinoma (OSCC). We found that there was overexpression of MELK in human OSCC tissues, and high MELK expression was correlated with lymphatic metastasis and led to poor prognosis in patients with OSCC. We also confirmed that MELK is closely correlated to the EMT process using a human OSCC tissue microarray. Additionally, MELK expression was observed to be regulated in several OSCC cell lines, and knockdown of MELK genes inhibited cell proliferation, migration, invasion and EMT of OSCC cells *in vitro*. Furthermore, silencing of MELK suppressed tumour growth *in vivo*, and experimental research verified that MELK may augment OSCC development via mediating the Wnt/Notch signalling pathway. Our findings suggest that MELK serves as an oncogene to improve malignant development of OSCC via enhancing EMT, and MELK might be a potential target for anticancer therapeutic.

## INTRODUCTION

Originating from the oral mucosal epithelium, squamous cell carcinoma is a lethal and deforming disease caused by tumour invasion, orofacial destruction, cervical lymph node metastasis and ultimate blood-borne dissemination [1]. There are 300,000 new cases reported worldwide each year, and the incidence has recently increased significantly, especially in young people [2]. Tobacco use, alcohol consumption and human papillomavirus (HPV) infections are generally considered risk factors for OSCC [3, 4]. Despite advances in treatment options for OSCC patients, the overall survival rate of OSCC is lower than 50%, nearly unchanged in the last three decades [3, 5]. It is therefore important to discover the mechanism underlying disease initiation and progression. Thus, there is an urgent need for find new biomarkers, especially molecular therapeutic targets for OSCC.

Epithelial to mesenchymal transition (EMT) is a developmental process in which epithelial cells acquire mesenchymal properties and is commonly seen at the invasive front of advanced tumours [6, 7]. Multiple studies have shown that EMT plays an important role in the invasive process of tumours [8, 9]. During EMT, cancer cells may indeed resemble cancer stem cells (CSCs) and exhibit enhanced self-renewal, metastasis and drug resistance capabilities [10, 11]. Multiple studies have also demonstrated that metastatic cancer cells, which probably undergo EMT, can exhibit a CSC phenotype [12, 13].

Maternal embryonic leucine zipper kinase (MELK) has been identified as a promising therapeutic target in multiple cancer types [14–16]. Emerging evidence has shown that abnormal expression of MELK might be involved in the tumorigenesis and progression of human cancers, such as prostate [17], breast [18], ovarian [19], oesophageal [20], gastrointestinal tract [21], and lung cancers [22], including chemotherapy resistance [14], stem cell renewal [14], and tumour growth [23]. Nevertheless, the underlying mechanism and biological significance of MELK, particularly in the cancer stem cells and EMT, are not yet clear.

In this study, we aimed to verify the expression of MELK in human OSCC tissue arrays. Furthermore, the correlation and role of MELK in EMT were investigated *in vitro* and *in vivo*.

## RESULTS

### Elevated MELK in human OSCC

**Figure 1:**
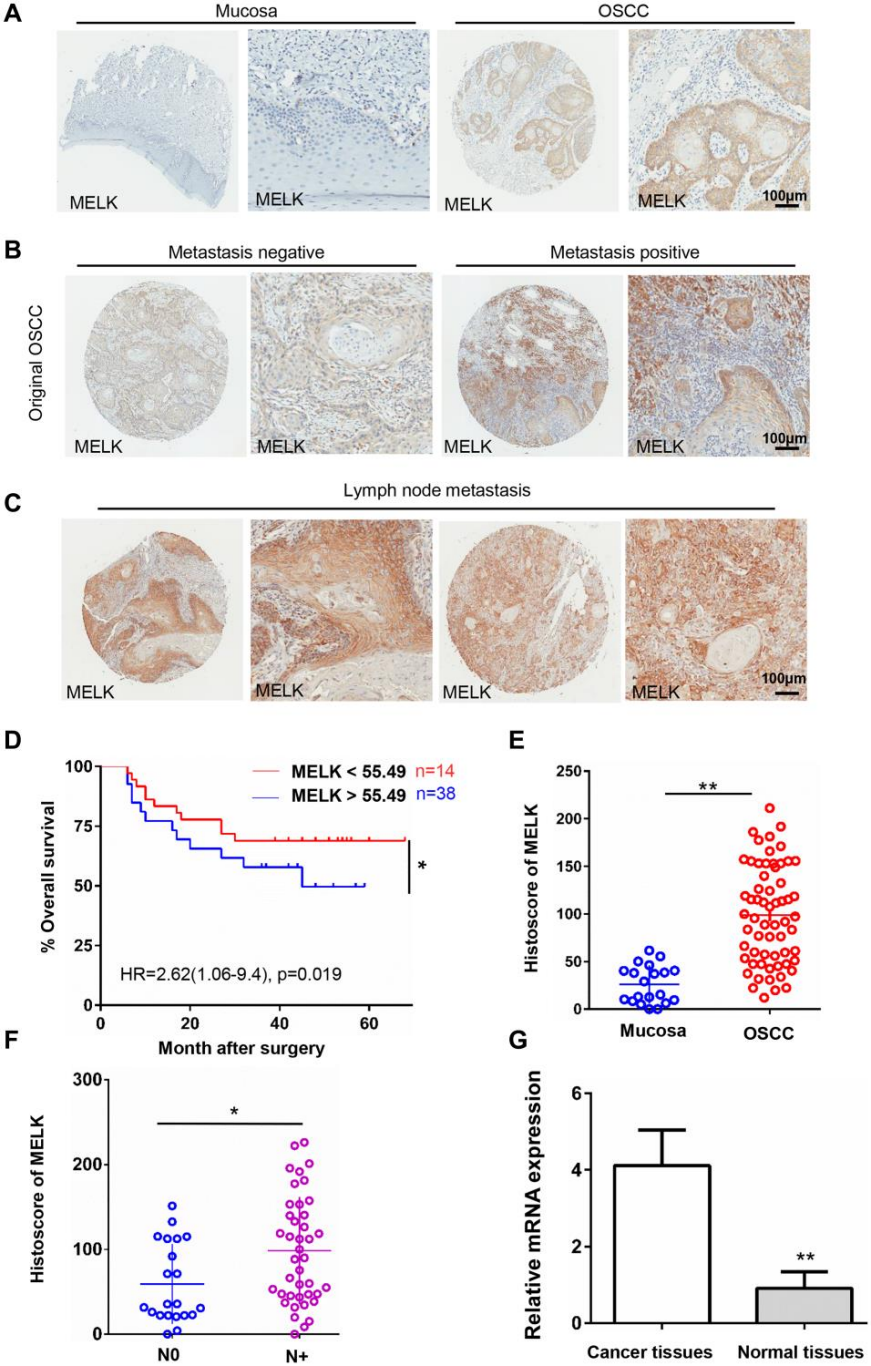
**High MELK expression in OSCC tissues correlates closely with lymph node condition.** (**A**) Representative images of MELK expression assessed via IHC staining normal oral mucosa (left) and OSCC (right). Scale bars = 100 μm. (**B**) Typical images of MELK expression assessed via IHC staining in metastasis-negative (left) and metastasis-negative OSCC (right). Scale bars = 100 μm. (**C**) Representative photographs of MELK in lymph node metastasis. Scale bars = 100 μm. (**D**) Kaplan–Meier analysis of OSCC patients with MELK expression level. (**E**) Score of MELK in oral mucosa (*n* = 20) and OSCC (*n* = 62). (**F**) Score of MELK in OSCC with or without lymph node metastasis. (**G**) The relative MELK mRNA level in patients with OSCC paired cancer and normal tissues. ^*^P < 0.05; ^**^P < 0.01; ^***^P < 0.001.

The expression of MELK in protein level was measured by IHC in our OSCC TMA. As shown in Figure 1A, 1E and 1G, the positive MELK staining was primarily located in the cytoplasm of cancer cells, and the expression in OSCC was significantly higher than that in normal mucosa tissues. MELK immunohistochemistry scores correlated significantly with the degree of pathological differentiation, with the poorer the pathological differentiation, the higher the staining score (Table 1). Next, we analysed the relationship between MELK and clinical pathological features, and the results suggested that the primary OSCC with lymph node metastasis (N+, *n* = 41) had higher immunoreactivity than the OSCC with non-metastatic lymph nodes (N0, *n* = 21, Figure 1B and 1F). Additionally, MELK level was increased in metastatic lymph nodes (Figure 1C). We next performed Kaplan–Meier analysis to study the prognostic value of MELK in OSCC, and log-rank analysis indicated the overall survival rate of patients with high MELK expression levels exhibited poor prognosis (Figure 1D). On the strength of these results, we considered that MELK expression might benefit in OSCC development, particularly in the metastatic characteristic to cancer stem cells.

**Table 1:** Statistical analyses of factors associated with survival in OSCC patients with the multivariate cox proportional hazards model.

**Variables**	**Overall survival**		***P* value**
	**RR**	**95% CI**	
MELK expression			
Positive VS negative	**2.657**	**1.246–5.146**	**0.006**
Gender			
Male VS female	0.713	0.342–1.834	0.662
Age			
<60 VS ≥60	1.763	0.422–2.486	0.542
Smoking			
Yes VS no	0.524	0.217–1.831	0.449
Drinking			
Yes VS no	1.344	0.623–2.813	0.341
Tumor stage			
3–4 VS 1–2	**3.321**	**1.663–5.681**	**0.008**
Lymph node metastasis			
+ VS –	**4.118**	**1.213–4.662**	**0.000**
Clinical stage			
3–4 VS 1–2	**2.227**	**1.413–4.14**	**0.000**
Histological type			
Poor VS well-moderate	1.317	0.712–1.334	0.519

Abbreviation: CI: confidence interval.

### MELK was closely associated with EMT in human OSCC

**Figure 2:**
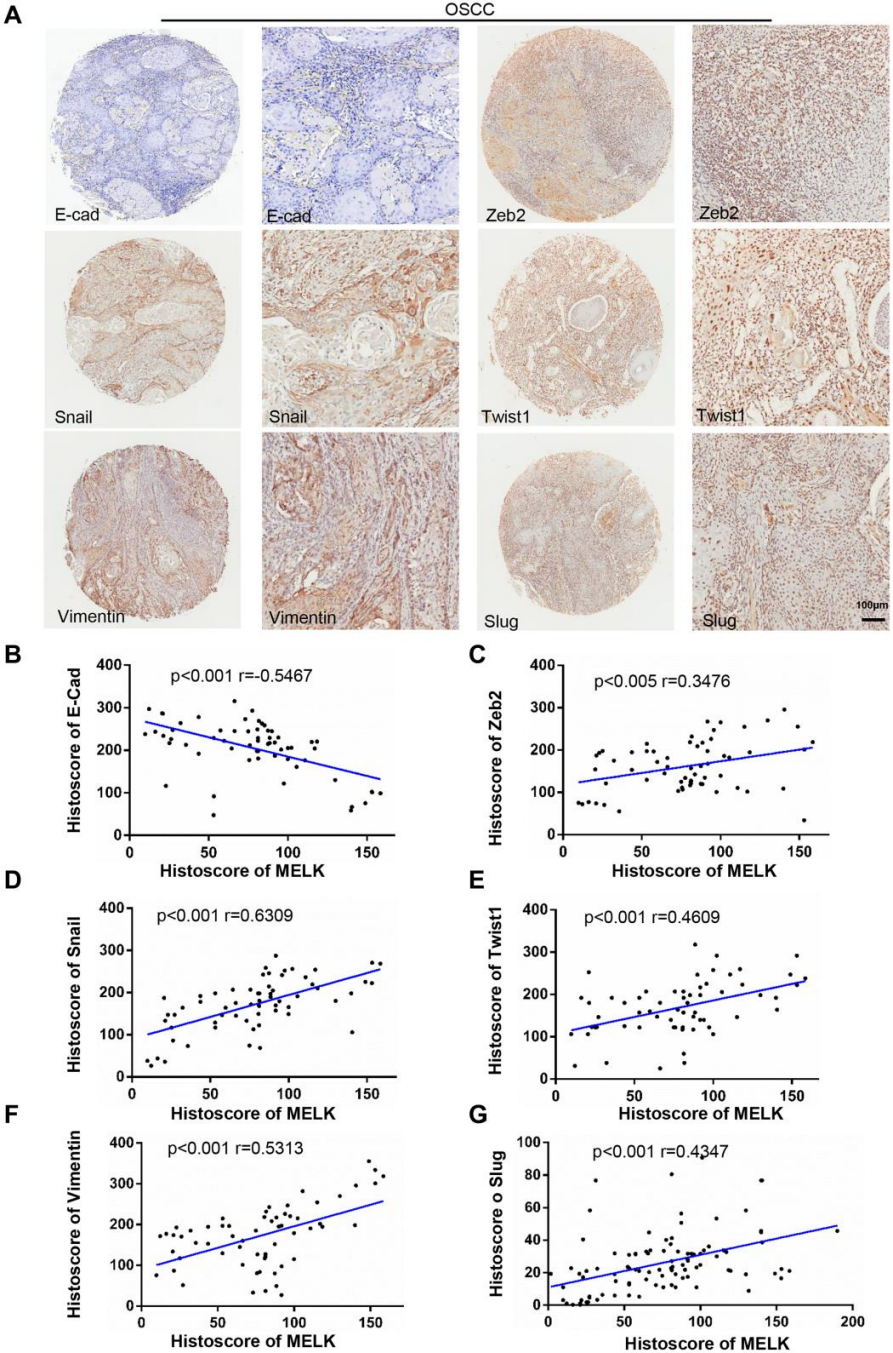
**Increased MELK expression was associated with EMT markers in human OSCC tissues.** (**A**) Typical images of IHC staining about N-cad, Zeb2, Snail, Twist1, Vimentin and Slug in human OSCC tissues. Scale bars:100 μm. (**B**–**G**) Correlation analysis among MELK and EMT-related proteins by Pearson’s correlation test based on the immunohistochemistry results.

EMT is well known to impel cancer recurrence and progression. To investigate if there is a correlation between MELK and EMT, the protein expression levels in human OSCC TMAs were examined using EMT markers (i.e., E-cadherin (Figure 2B), Zeb2 (Figure 2C), Snail (Figure 2D), Twist1 (Figure 2E), Vimentin (Figure 2F) and Slug (Figure 2G)); the results indicated that the mentioned proteins were highly expressed in OSCC tissues (Figure 2A). Afterwards, we were shocked to find that the expression of MELK in OSCC correlated remarkably with EMT markers. All the above findings suggest that MELK is a vital part in regulating EMT process in human OSCC.

### MELK knockdown suppresses OSCC cells growth

To further investigate the function of MELK in OSCC, the expression levels of MELK were measured in several OSCC cell lines, and using OKCs as negative control.

**Figure 3:**
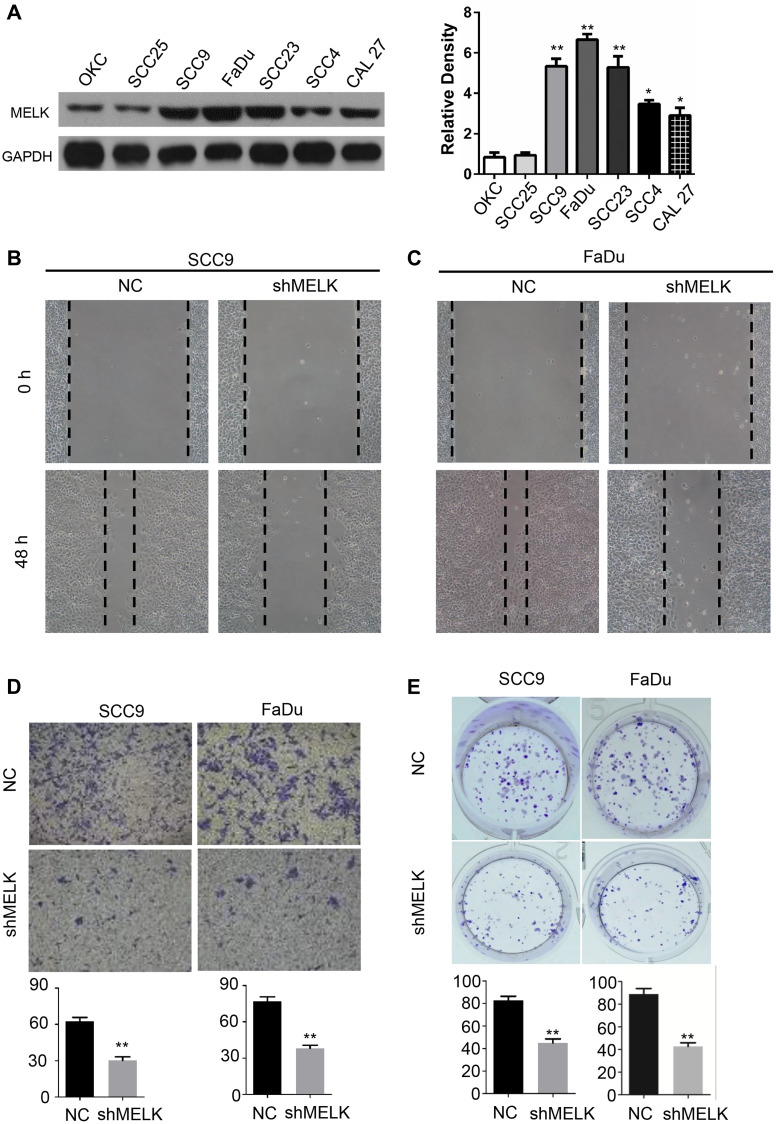
**MELK downregulation impedes growth of OSCC cells.** (**A**) Representative western blotting after transfection of shMELK in OKC and OSCC cell lines. Cell mobility of MELK knockdown in SCC9 (**B**) and FaDu (**C**) cell lines. (**D**) Iconic images and quantification of invaded cells. (**E**) Typical images and quantification of anchor-dependent colony formation. ^**^P < 0.01.

There was high expression of MELK in SCC9, SCC23, FaDu and CAL27 cell lines, and the highest expression level was presented in the FaDu and SCC9 cells (Figure 3A). Therefore, the FaDu and SCC9 cell lines were chosen for functional assays *in vitro*. shRNAs were performed to knock down MELK. In addition, as MELK expression is linked to lymph node metastasis in human OSCC, we conjectured that MELK may also motivate the migration and invasion of OSCC cell lines. Next, the most efficient shRNA sequence was used for wound healing and Transwell assays in FaDu and SCC9 cells. The ability of wound closure was decreased sharply after MELK knockdown for 48 hours (Figure 3B and 3C). In addition, similar finding was observed in the Transwell invasion assay (Figure 3D). Overall, the invasion ability of human OSCC cells was cut down validly by MELK knocking down *in vitro*. Moreover, MELK knockdown also reduced the number of anchor-dependent colony formation (Figure 3E).

### MELK and Notch and Wnt signaling pathways are positively correlated

**Figure 4:**
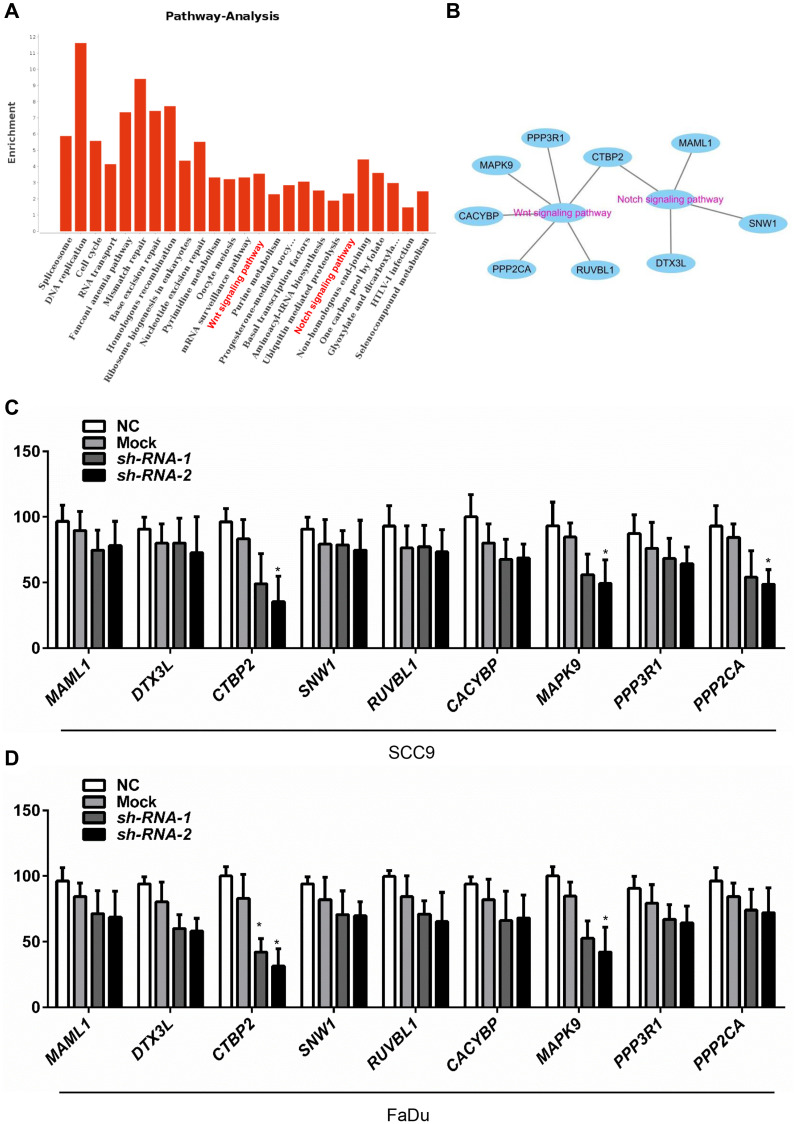
**MELK is positively correlated with the Notch and Wnt signalling pathways.** The essential enriched pathways of the MELK-correlated genes classified by KEGG analysis through DAVID (**A** and **B**). SCC4 (**C**) and Fadu (**D**) cells were transfected by shMELK, and Wnt/Notch signalling pathway-related gene expression was measured. ^**^P < 0.01.

The accurate pathways by which MELK may be regulated in human OSCC have not yet clear. To impartially identify the pathways associated with MELK, UALCAN using high-throughput RNA-sequencing data of the HNSCC cohort in the TCGA database was performed. Figure 4A and 4B showed that the mainly include enrichment pathways for MELK-correlated genes determined through KEGG analysis performed by DAVID. The MELK-correlated genes were enriched in mRNA surveillance pathways, cell cycle, Wnt signalling pathway, fanconi anaemia pathway, and Notch signalling pathways. Nevertheless, differentially expressed MELK-correlated genes were localized in the KEGG pathway and enriched in 2 peculiar pathways, containing the Notch signalling pathway and Wnt signalling pathway. To further confirm the effects of MELK on Notch/Wnt signalling pathways, we also detected the expression of Notch/Wnt signalling pathway-related gene via MELK knocking down. As shown in Figure 4C and 4D, the mRNA levels of key downregulated Notch/Wnt components, i.e., CTBP2 and MAPK9, were remarkably decreased after treatment with MELK shRNA in FaDu cells and SCC4 cells. Whereas regulators else of the Notch/Wnt signalling pathway in the KEGG analysis, for instance, CACYBP, PPP2CA, RUVBL1, DTX3L, PPP3R1, MAML1 and SNW1, were not significantly changed after treatment with MELK shRNA (Figure 4C–4D).

### MELK contributes to the EMT process in OSCC cell lines

**Figure 5:**
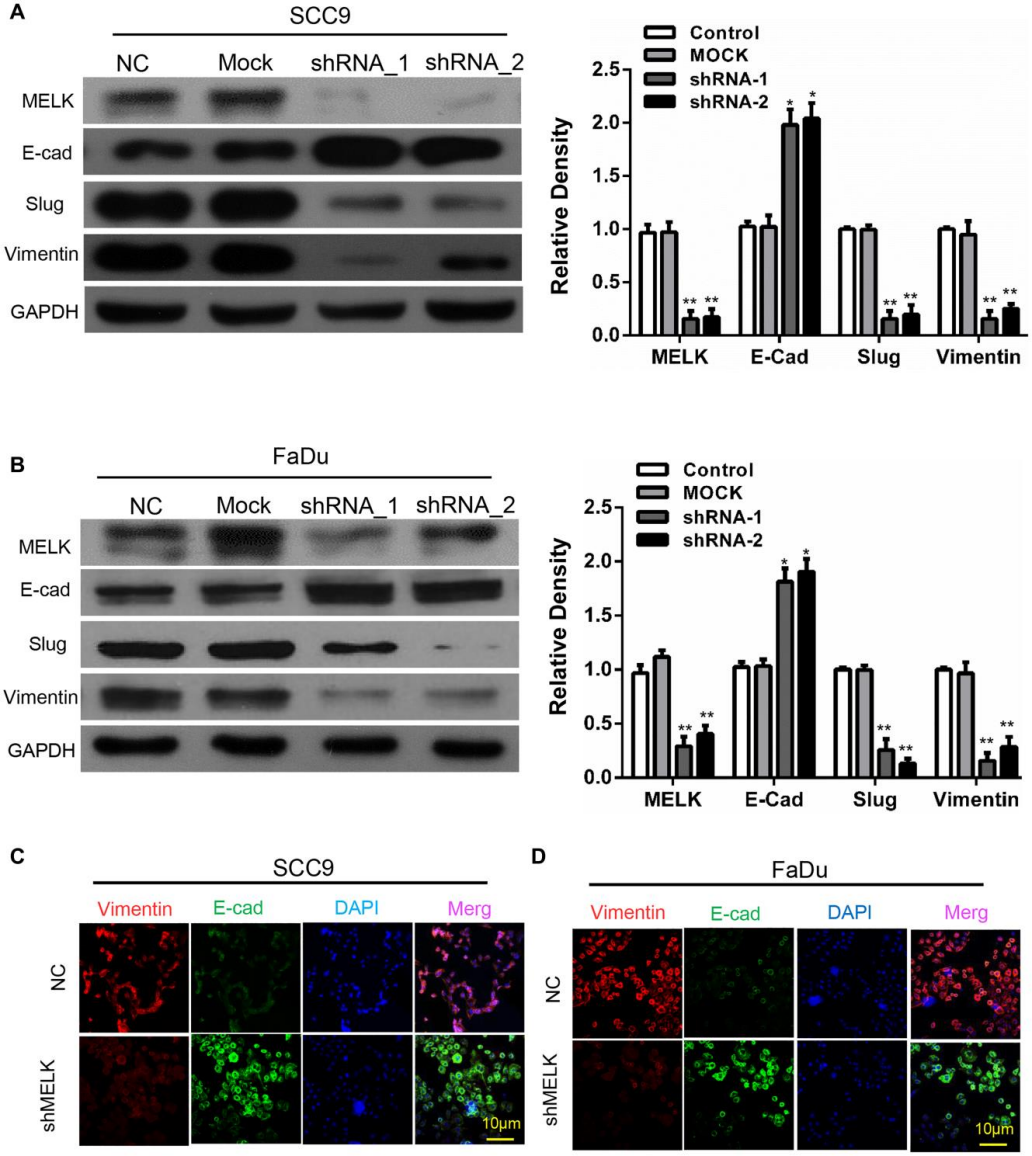
**Blocking MELK diminishes the EMT progress in OSCC cell lines.** Representative immunoblotting analysis of MELK, E-cad, Slug and vimentin expression after transfection of shMELK in SCC9 (**A**) and Fadu (**B**) cell. Immunofluorescence analysis of MELK, vimentin and E-cadherin in SCC9 (**C**) and FaDu (**D**) cells transfected with shMELK. Scale bars = 10 μm. ^*^P < 0.05, ^**^P < 0.01.

Next, to certify the shape of relation between MELK and EMT process, the EMT markers were measured by immunoblotting after MELK knocking down. Interestingly, the expression of N-cadherin, vimentin and Slug were significantly downregulated after MELK shRNA transfecting (Figure 5A–5B). Furthermore, immunofluorescence was used to detect the morphological expression in the cells. Similar results were noticed in SCC9 cells: N-cadherin and vimentin were decreased after knockdown of MELK (Figure 5C–5D).

### Downregulation of MELK restrains tumour proliferation, invasion and migration of OSCC *in vivo*

**Figure 6:**
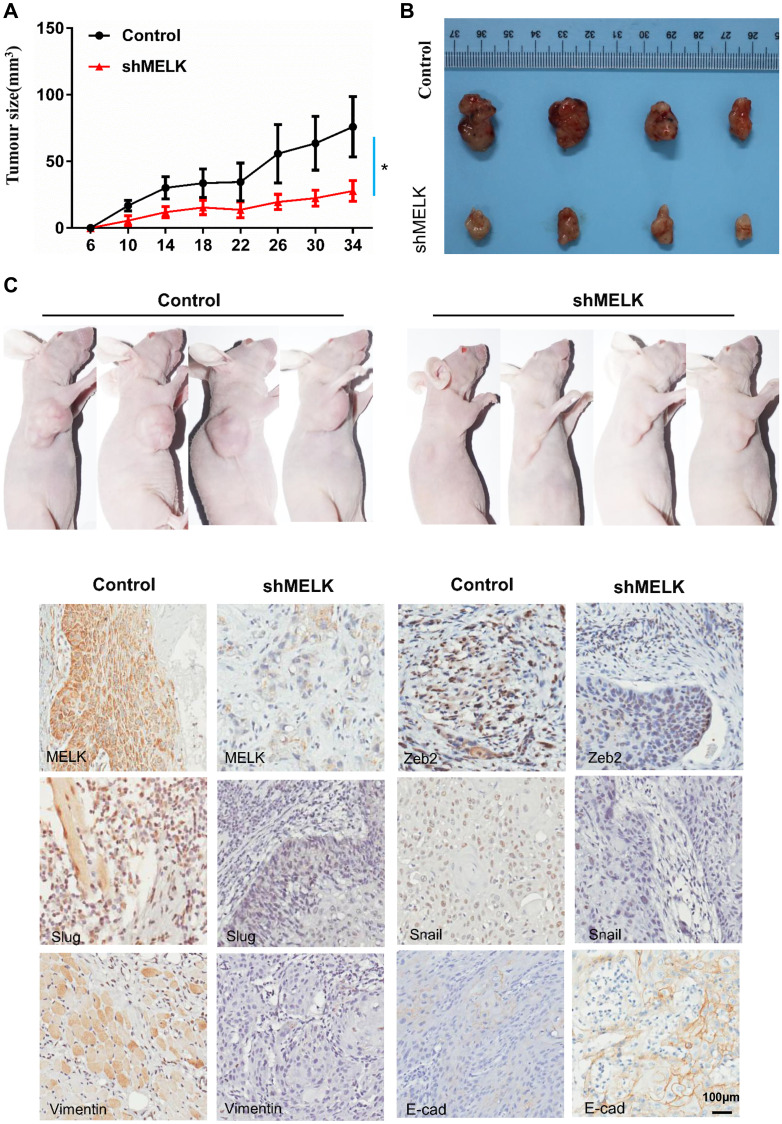
**MELK downregulation attenuates OSCC xenograft tumour growth in nude mice.** (**A**) Tumour growth curves for shMELK and NC mice. (**B**) Dissected tumours were photographed. (**C**) Representative images of IHC staining among MELK, Zeb2, Slug, Snail, Vimentin and E-cad in tumours; scale bar: 100 μm. ^*^P < 0.05, ^**^P < 0.01.

According to all the above findings *in vitro*, MELK was explored whether could promote the invasion and metastasis of OSCC *in vivo*. Establishment of a xenograft tumour model by subcutaneously injecting FaDu cells into nude mice. Inhibition of MELK sharply lessened tumour growth and decreased tumour volume (Figure 6A–6B). Moreover, IHC staining supported that MELK knockdown resulted in suppression of EMT markers (Figure 6C). Above all findings suggest that MELK exerts a vital regulatory function in EMT progress in human OSCC.

## DISCUSSION

Several researches have indicated that the EMT phenotype of tumours might exert a dominant function in carcinoma evolution and drug resistance [9, 12, 24, 25]. During the carcinoma progression, CSCs acquires mesenchymal features through EMT, migrates to adjacent mesenchymal tissues and invades into blood or lymph vessels [26]. However, little is known about the relationship between EMT and OSCC development. A comprehensive IHC analysis was performed to measure the expression of MELK in OSCC tissues. According to the results, we found that positive MELK staining was primarily located in the cytoplasm of cancer cells, and its expression was significantly higher in OSCC versus normal mucosa tissues. Subsequently, we observed we that OSCC with lymph node metastasis had a stronger immunoreactivity than OSCC without lymph node metastasis, and the expression of MELK was higher in metastatic lymph nodes than in the original tumour. In addition, the prognostic value of MELK in OSCC was investigated via Kaplan–Meier analysis. In line with our findings, the OS rate of Melk high expressing patients showed a poor prognosis. Additionally, EMT engaged in the process of cancer stem cells production, in which contribute to carcinoma development and chemoresistance [12, 27]. To explore the association between EMT and MELK in OSCC, we found that the expression vimentin, E-cadherin, Zeb2, Snail, Twist1 and Slug were markable correlated with MELK in OSCC. The above results shown that MELK may exert a momentous function in human OSCC during the EMT process. MELK knockdown lead to sharply reduce invasive and metastatic capacity in melanoma and lung cancer [28, 29]. In the current study, there was high expression of MELK in OSCC cell lines. Interestingly, the expression of EMT markers were markedly downregulated after MELK shRNA transfecting. Moreover, the self-renewal and ability of satellite stem cells were regulated by MELK [30]. Notably, the findings suggested that MELK downregulation dramatically weakened the migratory and invasive capacity. Thus, there may be an association between EMT and MELK. The above results indicated that MELK may exert a dominant function in the phenotype and maintenance of EMT in human OSCC. It could be latent target for therapeutic strategies for OSCC.

To further delve into the molecular mechanism by which MELK augments OSCC development, UALCAN analysis was performed to search for the genes associated with MELK in the HNSCC datasets of TCGA. For UALCAN, a weighted correlation network was established to find the most prominent module cooperate with MELK expression, which encompassed 238 genes. GO and KEGG enrichment analysis showed that pivotal module genes were chiefly enriched in mRNA surveillance pathways, cell cycle, Wnt signalling pathway, fanconi anaemia pathway, and Notch signalling pathways. Recent studies have shown that MELK can improve the development of renal cell carcinoma and bladder cancer through accelerating the EMT process [30]. Besides, the Wnt and Notch signalling pathways are vital in regulation of EMT [31, 32]. Therefore, the results of GO and KEGG enrichment analysis exhibited that EMT posed a pivotal capacity in the malignant process of MELK-regulated OSCC. GSEA hinted that the MELK high-expression group triggered more EMT gene sets than the MELK low-expression group. This is further evidence that EMT was regulated by MELK in OSCC. Our present study reveals that MELK might cause EMT to boost the malignant advancement of OSCC by altering cell adhesion.

In summary, the present study confirmed that MELK acted as an oncogene in OSCC development. Convincible evidences *in vitro* and *in vivo* reveal that MELK can accelerate OSCC progression by enhancing EMT process. These data provide valid strategies through suppressing or inactivating MELK in tumour tissues and might become a novel target for OSCC therapy.

## MATERIALS AND METHODS

### Ethics statement, patient specimens and human OSCC tissue microarray (TMA)

The acquisition of tumour samples from OSCC patients who were recruited in the present research was permitted by the Ethics Committee of Affiliated Hospital of Guilin Medical University. The proceedings for conducting human material are on the basis of the ethical requirement of the 1975 Declaration of Helsinki; the Declaration was last revised by the World Medical Association Congress in 2013. Original OSCC tissue specimens (*n* = 62) were gained from patients who endured surgery at the Department of Oral and Maxillofacial Surgery of Guilin Medical University, and tissue specimens of at least 3 cm of adjacent normal mucosa were obtained from the same group of patients as controls. The samples were collected from patients who did not accept chemotherapy prior to surgery from February 2010 to April 2011, and the survival patients had an average follow-up of ending in February 2015. Clinical staging and histological grading of OSCC according to appropriate guidelines. OSCC TMAs were contained with each patient’s specimen mentioned above and 1.5-mm tissue cylinders were performed by Shanghai Biochip Company, Ltd (Shanghai, China).

### Immunohistochemistry

Fixed tissues were buried in paraffin, sectioned to a thickness of 3 μm and immunohistochemical staining with a mouse anti-human MELK antibody (Santa Cruz Biotechnology, Santa Cruz, CA, USA, 1:100). Sections were prepared in mounting medium enclosing glycerol (Beyotime, P0126) and reflected by light microscope (Leica, DM6000M).

### Scoring system, hierarchical clustering

TMA sections were examined for background subtraction by Aperio ScanScope CS scanner (Vista, CA, USA) and evaluated (Version 9.1) for membrane, nuclear, or pixel quantification using Aperio Quantification software. Each tissue sample was scored based on the staining intensity (0, none; 1, weak; 2, moderate; 3, strong) multiplied by the percentage of stained cells (positive cells ≤25% of the cells: 1; 26–50% of the cells: 2; 51–75% of the cells: 3; ≥75% of the cells: 4). Calculated in the range 0–12. The median value of scores was engaged to verify the cut-off. Cancers scoring above the cut-off value were regarded as have expression of the mentioned molecule and vice versa.

### Quantitative real-time PCR

Total RNA was extracted using RNAiso (Takara, Japan) and reverse transcribed by the PrimeScript™ RT Reagent Kit with gDNA Eraser (Takara). Evaluation of relative levels of mRNA expression in line with the 2-ΔΔCT method.

### Cell culture

All cell lines were purchased from the Shanghai Cell Bank of the Chinese Academy of Sciences. SCC9, SCC15 and SCC25 cells were maintained in DMEM/F12 within 10% FBS and 400 ng/ml hydrocortisone, and Cal 27 and FaDu cells were cultivated in DMEM/high glucose with 10% FBS. The fundamental cultured OKC cells were retained in defined KSMF (Gibco BRL, Carlsbad, CA, USA). These cell lines were managed at 37°C in humidified atmosphere with 5% CO_2_.

### Generation of knockout cell lines

Transfecting with MELK shRNA was executed as stated in the manufacturer’s instructions. Briefly, FaDu and SCC9 cells were transfected with 1.4 μg/ml shRNA duplexes within Lipofectamine 2000 (Invitrogen). The human MELK sequences (5′-ccg ggc ctg aaa gaa act cca att act cga gta att gga gtt tct ttc agg ctt ttt g −3′ and 5′-aat tca aaa agc ctg aaa gaa act cca att act cga gta att gga gtt tct ttc agg c −3′) were confirmed by Sanger DNA sequencing (Source Biosciences, Berlin, Germany). Stable knock-down clones were collected and applied in subsequent studies.

### Wound healing assay

Cells were inoculated in 6-well plates at 100% integration. The wound gap was scratched by a 5-μl pipette tip after 10 h serum starvation. After three washes in PBS, serum-free medium was added. 4 randomly selected fields of each well were photographed after 3 h and 6 h under the microscope (Nikon Corporation, Tokyo, Japan). Later, the distance of wound extension was evaluated by Nikon Application Suite.

### Transwell assays

Transwell assays were measured by using Transwell chambers (Corning, Albany, NY, USA) as previous described. Transwell chamber coated with Matrigel (BD Biosciences) was used to detect Transwell invasion assay. SCC9 or FaDu cells were resuspended and seeded to the upper chamber containing medium without serum, and the lower chamber accommodated 10% FBS media. After regular treatment, the none-invaded cells were discarded and the invaded cells fixed, stained, photographed and countered.

### Immunofluorescence and confocal microscopy

Cells were planted in 10-mm glass-bottom dishes (Nest Biotechnology). After appropriate treatment, cells were fixed by 4% paraformaldehyde and permeabilized in 0.1% Triton X-100. Then, the cells were blocked and incubated with primary antibodies (N-cadherin and Vimentin, 1:200, CST, MELK, 1:100, Source Bioscience) and secondary antibodies. Nuclei were revealed by diamidino-2-phenylindole (DAPI). Capture fluorescent images under the laser scanning confocal microscope (Olympus).

### Bioinformatics analysis

UALCAN (http://ualcan.path.uab.edu/analysis.html) was performed to find the MELK-related genes in the HNSCC datasets of TCGA as previous described [33], and the DAVID functional annotation tools (https://david.ncifcrf.gov/) were performed to analyse the Kyoto Encyclopedia of Genes and Genomes (KEGG) pathway.

### Western blot analysis

Total protein from cells were extracted by RIPA buffer (Beyotime) and quantified by BCA Protein Assay Kit (Beyotime). Antibodies against human MELK (Source Bioscience, 1:1000), E-cadherin (CST, 1:1000), Slug (CST, 1:1000), and vimentin (CST, 1:1000) were used as primary antibodies.

### Mouse experiments

The animal administrations were permitted by the Animal Research Committee of the Experimental Animal Research Center of Guilin Medical University and in the light of the guidelines for international animal research. We established a subcutaneous tumorigenic model in nude mice as follows: male 1-month-old nude BALB/c mice (*n* = 6 per group) were subcutaneously injected with Cal27 (Scramble or sh-MELK) (1 × 10^6^) into both sides of buttocks of the mice. Tumours were measured every 4 days and the total tumour volume was calculated as V (volume, mm^3^) = 0.5 × L (length, mm) × W^2^ (width, mm^2^). After 6 weeks, all mice were sacrificed and subcutaneous tumours were resected and weighed.

### Statistical analysis

All data are presented as the mean ± SEM, and analysed using GraphPad Prism 5.01 (GraphPad Software, Inc., La Jolla, CA, USA) software. The differences in immunohistochemical staining were analysed via One-way ANOVA followed by Tukey’s post hoc or Bonferroni multiple comparison tests. Two-tailed Pearson correlation was used to analyse the relationship among MELK, Vimentin, E-cadherin, Zeb2, Snail, Twist1 and Slug. The TMA were determined by Mann-Whitney *U* test. All the experiments were executed at least three times independently, and the data are presented as the means ± SEM. *P* < 0.05 were considered to indicate statistical significance.
